# Supercritical Assisted Electrospray: An Improved Micronization Process

**DOI:** 10.3390/polym11020244

**Published:** 2019-02-02

**Authors:** Lucia Baldino, Stefano Cardea, Ernesto Reverchon

**Affiliations:** Department of Industrial Engineering, University of Salerno, Via Giovanni Paolo II, 132, 84084 Fisciano (SA), Italy; lbaldino@unisa.it (L.B.); scardea@unisa.it (S.C.)

**Keywords:** electrospray, supercritical CO_2_, atomization, microparticles, PVP

## Abstract

A new process is proposed that can largely improve classical electrospray (ESPR) atomization, thanks to the addition of supercritical CO_2_ (SC-CO_2_) to the liquid mixture, in which a polymer is dissolved, forming an expanded liquid. The consequent reduction of surface tension and viscosity allows the production of micrometric or nanometric particles of controlled size and distribution at a production rate up to one hundred times that of the traditional process. The new process was applied to particle generation from a very high molecular weight polyvinylpyrrolidone (PVP) and tested at different polymer percentages by weight and at different pressures. Repeatable microparticle diameters and distributions were obtained, ranging between 0.55 and 2.25 µm at PVP concentrations from 1 to 5% w/w and pressures between 80 and 120 bar.

## 1. Introduction

The production of micro- and nanoparticles with regular morphology and controlled size and distribution is a relevant target for many industrial fields, with pharmaceutical, biomedical, nutraceutical, energetics and fine chemical applications [1,2,3,4]. Therefore, several techniques have been proposed and used to produce these particles (e.g., microgrinding [5], micro- and nanoemulsions [6], spray drying [7], etc.). However, these traditional techniques suffer of some limitations, such as high process temperatures, shear stresses and large solvent residues [5,6,7].

One less conventional application to produce micro- and nanoparticles, is electrohydrodynamic atomization, also called electrospraying (ESPR) [2,4,8,9,10]. This technique has been proposed by several authors [4] to overcome the limitations of the traditional processes and has been especially proposed for polymers loaded with functional materials, active pharmaceutical ingredients and so on [2]. In ESPR, a solution containing a solute is fed through a nozzle. An electric potential, located between the nozzle tip and a ground electrode, influences the flow of the exit stream. The free charges located on the surface of the liquid tip develop electrostatic forces (Coulomb force) that, when a sufficiently high voltage is applied, compete with cohesive forces of the liquid (mainly surface tension and viscosity). When Coulomb force overcomes cohesive forces, droplets are formed and, operating at very small flow rates, it is possible to produce micro- or eventually nanoparticles. ESPR performance depends on many parameters, including applied voltage, distance between the injector and the ground collector, diameter of the nozzle, solvent volatility, electrical conductivity of the solution, solution viscosity, solution flow rate, temperature, humidity and surface tension of the solution, as well as the complex interplays among them. However, two process parameters are crucial in ESPR—the electrical potential and the liquid flow rate [9]—since these play a relevant role in processability of a solution and on the final size of particles [11].

However, this technique suffers from some limitations, specifically a difficulty in eliminating organic solvents during evaporation and a very limited range of operation (working window), mainly related to viscosity and surface tension of the solution to be processed. These limitations depend on physical parameters like kind and molecular weight of the polymer and concentration of the solution. In particular, when a critical limit of these parameters is reached due to an increase of surface tension and viscosity, liquid jet break-up (atomization) is not obtained and an unstable cone jet is formed [9,12], or fibers (instead of droplets) are obtained [13]. This last process is also well known and is named electrospinning (ESP); as a rule, ESPR is successful in a very narrow operating window, at very low solution flow rates. Using some reviews [2,4,9,14] and papers [8,10,12] as literature in the field of ESPR, capillary tips ranging between about 50 and 300 µm ID (internal diameter) have been used. Solution flow rates have generally ranged between 0.1 and 30 mL/h and the resulting microparticle diameter has usually comprised between about 2 and 200 µm. However, the same literature has also presented some cases in which, using very small solution flow rates of the order of magnitude of microliters/hour, nanoparticles have been produced down to about 50–100 nm [4,9,14].

Even when more evolved process configurations have been proposed, the limitation of the solution flow rate in ESPR has not been eliminated. For example, He et al. [15] proposed an integration of micromixer precipitation and ESPR that indicated the flow rate as the most important operating parameter affecting process efficiency and droplet size in ESPR. Curcumin nanoparticles were produced at 2 µL/h; sub-microparticles at 20 µL/h; and microparticles at 200 µL/h. Rezvanpour and Wang [16] proposed the addition of an auxiliary electric field in ESPR; poly(lactic-*co*-glycolic acid) solution flow rates between 1 and 2.5 mL/h were used, producing particles between about 3 and 6 µm and 6 and 12 µm, respectively. Ho and Lee [17] used ESPR in combination with spray drying to prepare poly(ethylene glycol)/polylactide core/shell microparticles. They verified that, operating in this manner, particle aggregation was avoided and improved core/shell structures were obtained, with a smaller particle size.

Solution viscosity and surface tension represent the key points in the transition between the formation of droplets and the formation of a continuous liquid phase. Viscosity depends on solvent/solute characteristics and on the concentration of solute/s. Surface tension is an intrinsic characteristic of the solvent due to interactions among liquid molecules located on the surface with ones inside the droplet. Therefore, both characteristics cannot be modified by traditional techniques. Moreover, being cohesive forces, they oppose liquid stream break-up and the consequent droplet formations that are at the basis of ESPR.

Supercritical fluid (mainly supercritical CO_2_, SC-CO_2_) based techniques have been proposed in the literature and in some cases in the industrial production of micro- and nanoparticles. Among them, supercritical antisolvent (SAS) [18,19], supercritical assisted atomization (SAA) [20,21] and supercritical emulsion extraction (SEE) [22,23] are the most used and have been successfully proposed to produce several micro-materials. SC-CO_2_ has been also used to assist electrospinning; indeed, some works have been proposed [24,25,26,27] in which fibers were spun in a pressurized vessel where SC-CO_2_ was flowing. The key point of those process arrangements was the contact between SC-CO_2_ and the forming fibers in the high-pressure vessel.

Polyvinylpyrrolidone (PVP) is a polymer approved for pharma, biomedical and food applications; it has been tested by some authors to produce micro- and nanoparticles through ESPR. Yu et al. [28] used PVP (*M*_w_ 58,000) and ketoprofen to produce polymer-drug composites through ESPR. 1 mL/h was the flow rate electrosprayed using a metal needle of 500 µm ID. Nanoparticles in the 320–640 nm range were obtained, depending on drug loading. Wang et al. [29] proposed a drug delivery system formed by chitosan nanoparticles as the core and PVP (*M*_w_ 50,000) micro/nanocoating as the shell, produced by ionic gelation and emulsion electrospray methods at a flow rate of 1.5 mL/h. Naproxen and rhodamine B were encapsulated in the core and shell regions, respectively. These authors obtained particles down to about 550 nm mean diameter. Kawakami et al. [30] adopted a coaxial electrospray using an inner and an outer nozzle of 0.4 and 0.8 mm, respectively, to produce a particulate core–shell solid dispersion of poly(methacrylic acid-*co*-methyl methacrylate) (Eudragit L-100) as the shell and PVP (*M*_w_ 10,000) K12-17 in the core phase. However, particles with an irregular morphology were obtained. Yu et al. [31] electrosprayed PVP and acetaminophen, obtaining sub-microparticles with an average diameter of 0.88 ± 0.17 µm.

In order to overcome the limitations of classical ESPR, it could be possible to introduce a process in which viscosity and surface tension of the solution can be reduced. These forces are characteristic of the fluid used, but they could be modified by adding large quantities of gas solubilized in the liquid solution. Indeed, the massive presence of a gas dissolved into a liquid can largely reduce its viscosity and surface tension [32]. An expanded liquid is formed, in which the presence of solubilized gas molecules strongly reduces surface tension because they interpose among the solvent molecules in the liquid phase, largely reducing their interactions and interactions with those molecules located on the liquid surface [32]. If sufficiently large quantities of SC-CO_2_ are dissolved in a liquid, the expanded liquid that is formed can even show near-zero surface tension. The presence of SC-CO_2_ also largely reduces viscosity, since CO_2_ small molecules can interpose among the polymer ones favoring the mobilization of large polymeric chains.

Following this line of reasoning and the analysis of the previous literature, a new technique is proposed for the first time in this work, named supercritical assisted electrospraying (SA-ESPR). In SA-ESPR, expanded liquid polymeric solutions are formed by dissolving controlled quantities of SC-CO_2_ in a liquid solution and then the expanded liquid is electrosprayed instead of the ordinary solution in a chamber operated at atmospheric pressure. It is worth noting that in previous works [24,25,26,27], when SC-CO_2_ has been coupled with electric fields, fibers have been formed. In such instances, electrospinning was obtained and SC-CO_2_ was used only as a viscosity reducer during fiber spinning in the high-pressure vessel. It is expected that, using this new process arrangement, high molecular weight polymers, larger liquid flow rates and a large range of polymer concentrations could be used, with the possibility of producing smaller particles and a faster solvent elimination due to the contribution of SC-CO_2_ during drying. In order to demonstrate the capabilities of this process, a high molecular weight PVP has been used (*M*_w_ 1,300,000), which has dissolved in ethanol (EtOH) at various weight concentrations. Different injection pressures have also been tested.

## 2. Apparatus, Materials and Methods

Polyvinylpyrrolidone (*M*_w_ 1,300,000) and ethanol (purity > 99.9%) were bought from Sigma Aldrich. CO_2_ (purity 99.9%) was supplied by Morlando Group s.r.l. (Sant’Antimo, NA, Italy). PVP powder was dissolved in ethanol at different concentrations by weight (ranging from 1 to 5% *w*/*w*), at room temperature and using a magnetic stirring.

**Figure 1:**
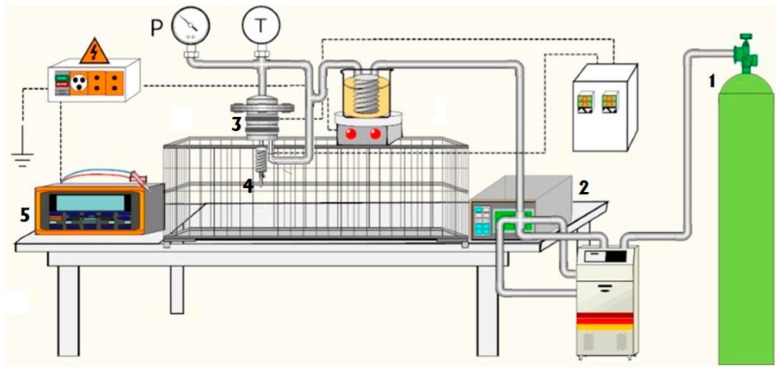
Schematic representation of the supercritical assisted electrospraying (SA-ESPR) plant: (1) CO_2_ storage tank, (2) high-pressure pump, (3) high-pressure vessel, (4) nozzle and (5) generator.

The SA-ESPR apparatus mainly consisted of a stainless-steel high-pressure vessel with an internal volume of 60 mL (feeding vessel), in which 50 mL of a PVP–ethanol solution (at different PVP concentrations by weight) were loaded. The vessel was closed and liquid CO_2_ was pumped from the bottom using a high-pressure pump (Gilson, mod. 305, Middleton, WI, USA) up to the desired pressure, to promote mixing with the liquid polymeric solution. The scope was to allow the obtainment of a near-equilibrium solution when SC-CO_2_ was added to the vessel, which, from the point of view of the binary mixture CO_2_ and ethanol, can be classified as an expanded liquid. When process pressure was reached, the system was left 10 min for equilibration, allowing the SC-CO_2_–polymeric solution mixture formation; this equilibration time was selected after feasibility tests which showed that times larger than 10 min did not produce any detectable variation in jet behavior, particle size or particle size distribution (PSD). Nitrogen was introduced at the same pressure selected for the process from the top of the vessel, to avoid pressure modification during microparticle production; it acted as a sort of piston to favor polymeric solution flow when a valve (Swagelok ON/OFF), located at the bottom of the feeding vessel, was opened to start the experiment. In particular, when the valve was opened, a pressure gap was generated between the operative pressure of the vessel and the room conditions at the exit of the injector. Therefore, in order to obtain a uniform flow of the polymeric solution, the use of nitrogen as a piston was required. SA-ESPR was performed at 35 °C and at different pressures (80, 100 and 120 bar) for about 15 min. Pressure in the vessel was measured by a test gauge (mod. MP1, OMET, Lecco, Italy); temperature was regulated using a proportional–integral–derivative (PID) controller (mod. 305, Watlow, St. Louis, MO, USA). The generator (FUG Elektronik, mod. HCP 35-3500, Schechen, Germany) was set at 30 kV and the injector–collector distance was fixed at 25 cm. The collector was, as a rule, formed by two adjacent stainless-steel blocks, covered by a thin aluminum foil and isolated from the external environment by a plexiglass hollow cylinder with an internal diameter of 30 cm. However, different configurations were tested, as proposed in Results and Discussion section. During processing, the solution flowed in a short stainless-steel tube (Swagelok, 1/4 inch), and was heated with a spiraled resistance (275 W, Watlow, St. Louis, MO, USA) to avoid freezing of the solution as a result of the Joule–Thomson effect produced by CO_2_ expansion. A 140 µm ID thin wall nozzle was used. The experiment ended when the whole content of the feeding vessel was discharged. Each experiment was repeated twice. A sketch of the laboratory plant is presented in Figure 1.

PVP particles were collected on an aluminum stub and sputter coated with gold (Agar Auto Sputter Coater mod. 108 A, Stansted, UK) at 30 mA for 80 s and analyzed by a field emission scanning electron microscope (FE-SEM, mod. LEO 1525, Carl Zeiss SMT AG, Oberkochen, Germany) to study their morphology.

Sigma Scan Pro 5.0 (Jandel scientific, San Rafael, QC, Canada) and Origin 9.1 (Microcal, Northampton, MA, USA) were used to determine the average diameter of the particles and to calculate particle size distribution using different SEM images. Approximately 300 particle diameters were measured for each distribution.

Ethanol residue was measured by a headspace (HS) sampler (mod. 7694E, Hewlett Packard, Palo Alto, CA, USA) coupled with a gas chromatograph (GC) interfaced with a flame ionization detector (GC-FID, mod. 6890 GC-SYSTEM, Hewlett Packard, Palo Alto, CA, USA). Ethanol was separated using two fused-silica capillary columns connected in series by a press fit, with the first column (mod. Carbowax EASYSEP, Stepbios, Bologna, Italy) connected to the detector (30 m length, 0.53 mm ID, 1 μm film thickness), and the second (mod. Cp Sil 5CB CHROMPACK, Stepbios, Italy) connected to the injector (25 m length, 0.53 mm ID, 5 μm film thickness). GC conditions were described in the USP 467 Pharmacopoeia with some minor modifications—the oven temperature was raised from 45 °C to 210 °C for 15 min. The injector was maintained at 135 °C (split mode, ratio 4:1), and helium was used as the carrier gas (5 mL/min). Headspace conditions were: equilibration time, 30 min at 95 °C; pressurization time, 0.15 min; and loop fill time, 0.15 min. Headspace samples were prepared in 20 mL vials filled with 3 mL of an internal standard (1,2-dimethylimidazole), and 500 mg of NaCl and water (0.75 mL) in which samples of PVP particles were suspended.

## 3. Results and Discussion

Before starting the description of the experimental results, it is opportune to try to characterize the fluid system formed in the feeding vessel. The initial liquid solution of ethanol and PVP, at atmospheric pressure, transformed progressively in an expanded liquid due to SC-CO_2_ loading and pressure increasing. To understand this, it is necessary to consider the binary system that has been extensively studied [33]. During CO_2_ loading, gas dissolves in the liquid that can largely expand (CXL: CO_2_ expanded liquid) and fill the whole volume of the feeding vessel. Indeed, given the internal volume of the feeding vessel at 60 mL, and an initial EtOH loading of 50 mL, a volumetric expansion of only 20% is enough to allow CLX to fill the whole volume (i.e., a relatively small quantity of CO_2_ is required to obtain this result). CXL can show expansion much larger than 100%, a behavior that can be generalized for all commonly used organic solvents, including ethanol [34]. That said, an expansion of 20% can be obtained at pressures around 10 bar; correspondingly, a x_CO2_ of about 0.1 is required.

In this work, however, ternary mixtures with 1% *w*/*w* to 5% *w*/*w* PVP were used and, correspondingly, the mixture critical point (MCP) of the ternary mixture was located at pressures larger or smaller than that of the binary system. An anti-solvent effect could be observed in the feeding vessel, due to the possible reduction of PVP solubility in EtOH in the presence of CO_2_. It is possible that PVP precipitation can block the injector at the bottom of the vessel, but this possibility was avoided in this case through the very large solubility of PVP in EtOH, and also because the formed CXL contained a relatively small percentage by weight of CO_2_. Therefore, the sequence of operation was pressurization of the solution, solubilization of SC-CO_2_ in it and injection through a small nozzle. This last step can induce atomization without the help of the electric field; therefore, some preliminary experiments were performed where the electric potential was set at zero. In the range of experimental conditions explored in this work, no atomization of the solution was observed, and the liquid was pulled out from the injector as a continuous liquid flow. Having ascertained that the SC-CO_2_ addition alone was not capable to atomize such solutions, all the experiments that followed were performed by activating the electric field, set at 30 kV, which is the most-used value of this parameter in the literature [35]. Systematic experiments were performed using EtOH solutions containing 1, 2, 3 and 5% *w*/*w* PVP and operating at 80, 100 and 120 bar. The results are summarized in Table 1.

**Figure 2:**
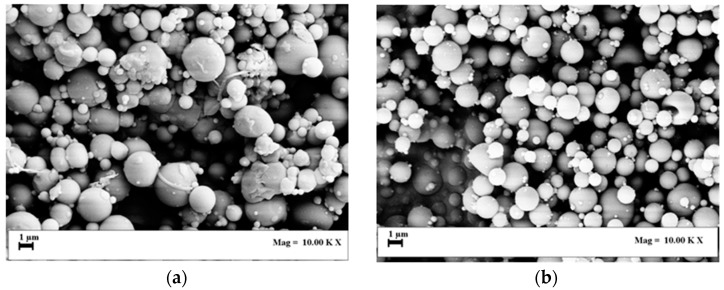
SEM images of PVP (1% *w*/*w*) particles produced at (**a**) 80 bar and (**b**) 120 bar.

At all tested operating conditions, SA-ESPR was successful in producing spherical microparticles and no polymer precipitation was observed in the feeding vessel. For example, the two SEM images reported in Figure 2a,b show the microparticles recovered on the collector at a PVP concentration of 1% *w*/*w* and pressures of 80 and 120 bar. These give a qualitative indication of particle diameter reduction as a result of increased operative pressure, and provide information about their spherical non-coalescing morphology.

**Figure 3:**
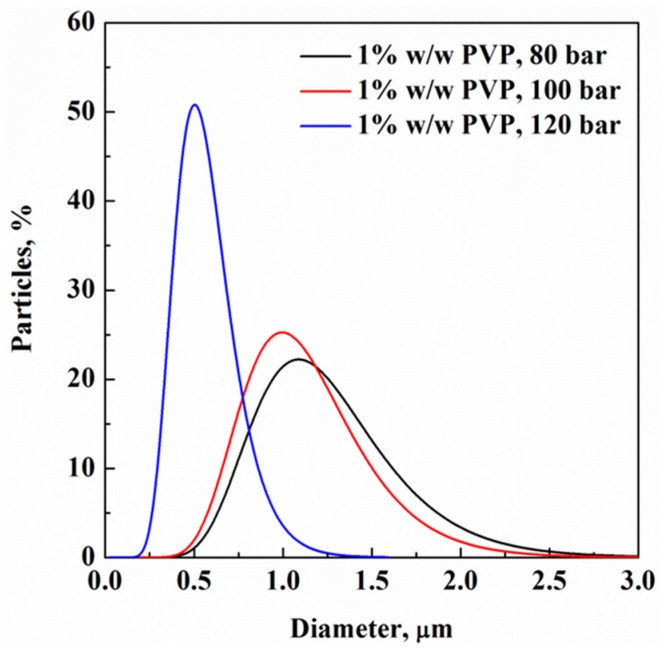
Particle size distributions (PSD) of 1% *w*/*w* PVP particles produced at different pressures.

Particle size distributions (PSD) for 1% *w*/*w* PVP experiments were produced using specialized software, as previously indicated in Apparatus, Materials and Methods, and are reported in Figure 3. A clear trend can be identified: at the same concentration as the liquid solution, an increase of SA-ESPR injection pressure induces the formation of smaller particles, and their PSD becomes narrower. For example, at 120 bar, the mean particle diameter is 0.55 µm and the standard deviation is 0.21 µm; i.e., near monodisperse nanoparticles were produced. Operating at 80 bar, mean diameter becomes 1.20 µm and standard deviation is 0.44 µm. The other distribution data are reported in Table 1.

**Figure 4:**
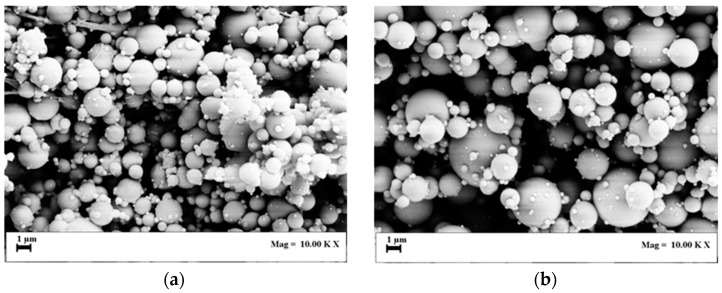

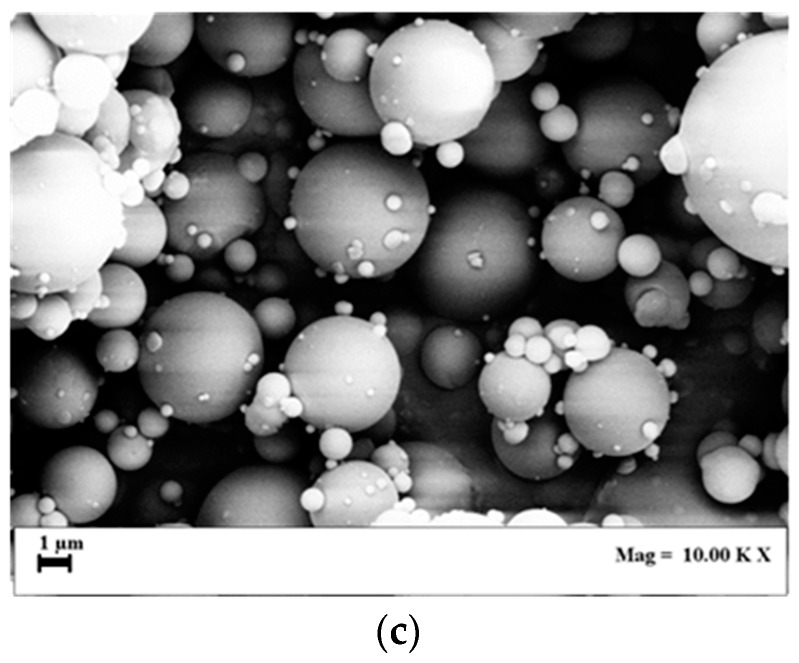
SEM images of PVP particles produced at 120 bar: (**a**) 2% *w*/*w* PVP, (**b**) 3% *w*/*w* PVP and (**c**) 5% *w*/*w* PVP.

Other sets of experiments were performed using solutions at 2, 3 and 5% *w*/*w* PVP, since it is known from the literature that polymer concentration can largely modify droplet size and distribution [36]. For example, SEM images of the experiments performed at 120 bar are reported in Figure 4a–c. In all these cases, particles were spherical, but they are qualitatively larger than those obtained at 1% *w*/*w* PVP. In particular, operating at 3% *w*/*w* PVP, mean particle diameter was 1.11 µm at 120 bar and 1.60 µm at 80 bar. Again, all distribution data is reported in Table 1.

**Figure 5:**
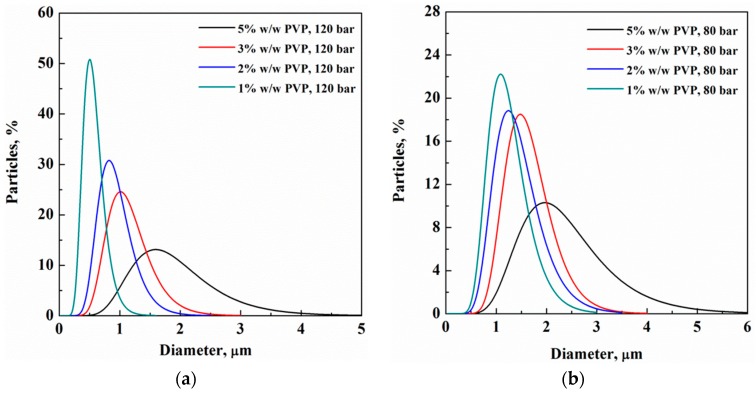
PSD of PVP particles obtained at 1, 2, 3 and 5% *w*/*w*, working at (**a**) 120 bar and (**b**) 80 bar.

The analysis of the particle diameter observed at fixed pressure and at different PVP concentrations produced diagrams like the ones reported in Figure 5a,b, where the results obtained at 120 bar and 80 bar are collected. It is evident that PVP particle size reduced, decreasing the polymer concentration in the starting solution in both cases. The mean particle size reduced at higher pressures and the corresponding PSD also became narrower.

**Figure 6:**
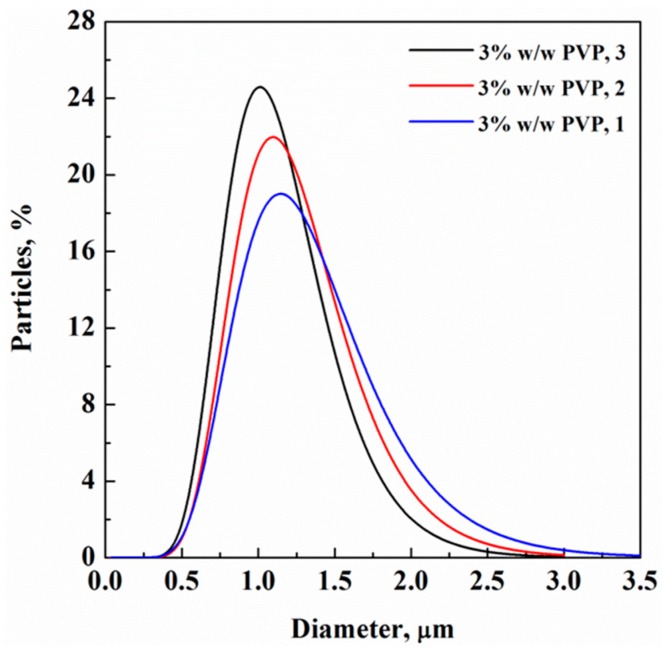
PSD of PVP particles at 3% *w*/*w* collected on (1) stainless-steel blocks in a plexiglass delimiter, (2) stainless-steel blocks and a metallic spiral and (3) stainless-steel blocks and a metallic spiral in a plexiglass delimiter.

At this point in the work, a further series of experiments was performed to evaluate the possible influence of collector geometry on particle collection, since some authors indicated this part of the apparatus as possibly involved in producing different PSDs during ESPR [37]. Besides the collector geometry described in the Apparatus, Materials and Methods section, another geometry was proposed: the addition of a metallic spiral on top of the stainless-steel blocks, covered with aluminum foil. An example of the different PSD obtained by changing the collector configuration is reported in Figure 6 for a PVP concentration of 3% *w*/*w* and operating at 120 bar. Mean particle sizes between 1.18 and 1.11 µm (mean variation about 6%) were observed at a nearly constant PSD value. The observed differences largely fell within the calculation error for producing distributions based on the SEM images. Therefore, it is possible to conclude that the collector had a negligible role in the experiments performed.

The observed effects of SA-ESPR process parameters on PVP particle size are relatively simple to explain. An increase in operating pressure means that a larger quantity of CO_2_ is dissolved in the polymer, and the corresponding expanded solution will be characterized by less surface tension [32]. Moreover, larger pressures also mean a larger pressure drop of the injected solution. Both of these effects can reduce the mean particle size of the particles produced. A measure of the relevance of surface tension in SA-ESPR is given by a dimensionless characteristic, the Weber number, defined as (ρv^2^L/σ) where ρ is the solution density, v is the velocity, L is the droplet diameter and σ is the surface tension. The Weber number gives a measurement of the relative importance of fluid inertial forces with respect to surface tension; it is, therefore, useful to analyze the formation of droplets and bubbles. Larger Weber numbers indicate that inertial forces overcome cohesive forces, and an increase of pressure produces an increase of disruptive forces which tend to reduce droplet diameter, as in classical atomization processes like spray drying. The Weber number, however, does not take into account the influence of the electric field on disruptive forces, in the event of ESPR processes.

In these experimentations, the presence of CO_2_ exiting from the expanded solution during atomization also favored organic solvent (EtOH) elimination. Indeed, solvent residue analyses were also performed and EtOH values lower than 5 ppm were found for all PVP particles generated.

A final consideration is related to particle collection: As in ESPR, the SA-ESPR process took advantage of the fact that electrically loaded particles repel each, other avoiding coalescence phenomena, while they were attracted by the collector and blocked on its surface.

## 4. Conclusions and Perspectives

The new SA-ESPR process maintained its promises, and further parameters can be varied with respect to traditional ESPR; namely, surface tension and viscosity can be largely reduced. These conditions improve the process and produce a wide enlargement of its operating window—a very high molecular weight PVP (*M*_w_ 1,300,000) was successful processed, whereas only molecular weights of 400,00 and 55,000 were generally used in the previous literature. This indicates that solution flow rates up to two or three orders of magnitude larger can be used with respect to traditional ESPR, allowing the production of large particle batches—a result that is relevant to industrial production. Nano- and microparticles with mean diameters ranging between 0.55 and 2.25 µm were obtained with a good repeatability and very narrow PSDs, especially operating at 120 bar. EtOH is one of the safer solvents from the point of view of toxicity; therefore, future experiments that involve the addition of pharmaceutical active principles will be favored. Moreover, attempts to produce even smaller particles could be also performed using different polymers and coupled polymer-active principles.

## Figures and Tables

**Table 1:** Mean particle diameter and standard deviation for the experiments performed in this study. PVP: polyvinylpyrrolidone.

Mean particle diameter, µm	PVP concentration			
Pressure, bar	1% *w*/*w*	2% *w*/*w*	3% *w*/*w*	5% *w*/*w*
80	1.20 ± 0.44	1.38 ± 0.56	1.60 ± 0.68	2.25 ± 1.38
100	1.09 ± 0.43	1.14 ± 0.55	1.30 ± 0.55	2.03 ± 1.19
120	0.55 ± 0.21	0.90 ± 0.40	1.11 ± 0.50	1.81 ± 0.82
